# TV Series in Mainstream Media Depicting Autism and Self-Diagnosis of Autism in a General Population of Young Adults

**DOI:** 10.1007/s10803-023-06150-z

**Published:** 2023-10-17

**Authors:** Gloria Mittmann, Beate Schrank, Verena Steiner-Hofbauer

**Affiliations:** 1 Research Centre Transitional Psychiatry, Karl Landsteiner University for Health Sciences, Krems, Austria; 2 Department of Psychiatry and Psychotherapeutic Medicine, University Hospital Tulln, Tulln, Austria

**Keywords:** Autism, Diagnosis, ASD, Media portrayal, Social contagion

## Abstract

**Purpose:**

The prevalence of autism diagnoses has increased in recent years. The portrayal of autistic characters in mainstream media, such as TV series, may be a contributing factor. This study investigated whether young adults who consume media featuring autistic characters are more likely to self-diagnose with autism.

**Methods:**

348 participants filled out an online questionnaire exploring their media consumption, subjective diagnosis of autism and objective indicators of autism using an Emotion Recognition Task.

**Results:**

Results from linear regression analysis indicated a significant correlation between media consumption and self-diagnosis, while valence of the series and objective diagnosis did not have a significant influence. The study found no gender differences.

**Conclusion:**

The results suggest a need for further research on the relationship between media consumption and self-diagnosis, including for other forms of media beyond TV series.

## Introduction

Autism is a neurodevelopmental condition that is characterised by repetitive behaviours and difficulties in communication and social interactions (World Health Organization, 2019). According to two recent systematic reviews, the prevalence of diagnoses has increased in recent years for both children (Zeidan et al., 2022) and adults (Huang et al., 2020). This increase is attributed to better reporting practices (Hansen et al., 2015), changes to diagnostic criteria (World Health Organization, 2019), detection and identification, as well as increased awareness about the condition not only amongst professionals but also in the general population (Huang et al., 2020; Zeidan et al., 2022).

Especially for adults, this higher awareness might in parts stem from an increase in the depiction of autistic characters in mainstream media. In recent years, many TV series have been prominently featuring characters with autistic characteristics. Literature suggests that the representation of autistic characters can have an influence that can both benefit and harm opinions about autism in a general population (John et al., 2018; Nordahl-Hansen & Øien, 2021; Nordahl-Hansen, Tøndevold et al., 2018). For example, portrayal often lacks diversity and characters are primarily male with savant syndrome (Dean & Nordahl-Hansen, 2022; Mittmann et al., 2023; Nordahl-Hansen, Øien et al., 2018). Yet, portrayal can also lead to more appreciative awareness and understanding of autism and the specific characteristics and challenges (Fontes & Pino-Juste, 2022; Nordahl-Hansen & Øien, 2021).

Furthermore, adults are often underrepresented in research around autism. Many autistic adults go undiagnosed or are only self-diagnosed (Huang et al., 2020; McDonald, 2020) and there is a lack of health services for autistic adults in general (Huang et al., 2020). This is specifically important for young adults to help in the time of transition from youth to adult health care (Murphy et al., 2016), a time in which there are prominent social challenges and changing communicative requirements (Eccles et al., 2003). In terms of diagnosing, autism in adulthood often only gets recognised when it coincides with other mental illnesses, which can then lead to wrong diagnoses (Kamio et al., 2013).

While a lot of focus has been put on how portrayal of autistic characters can influence stigmatisation and awareness, less research has investigated how it influences the perceptions of one’s own characteristics. Recent literature in the area of social media suggests that new media can have a highly relevant influence on self-perceived symptoms and diagnoses (Haltigan et al., 2023): Online environments can lead to a subjective “identity-selection”, especially when it comes to tic-like behaviours such as Tourette syndrome (Frey et al., 2022). But social media has also led to concerns about self-diagnosing other mental conditions, ranging from eating disorders to bipolar or borderline disorders (Haltigan et al., 2023). Importantly, all these phenomena rely on self-diagnosis rather than professional objective diagnoses. The authors point out that there is an urgent need for more research in this area.

While self-portrayal on social media and the phenomenon of identity-selection in this online environment has elicited some attention, other media forms could also play a role in self-diagnosis. In terms of media consumption, young adults may relate to the characters they see on screen in mainstream series. Identification with fictional characters describes a phenomenon where viewers may adopt a character’s viewpoint, goals or mental states (Broom et al., 2021; Cohen, 2018). Considering many adults have not been diagnosed as children, increased media portrayal of autistic characters might therefore lead to more self-diagnoses due to higher understanding and awareness. With so many series available for consumption by young adults, this study sought to investigate how TV shows that depict autism or autism-like symptoms may influence self-diagnosis of this condition. Therefore, our primary research question was if there is an association between media consumption and self-diagnosis of autism. Our secondary aim was to investigate the influence of objective diagnosis and subjective valence of the series and characters on this link.

## Methods

### Recruitment and Study Procedure

The study was conducted via a cross-sectional online questionnaire. After covering sociodemographic questions (age, gender and nationality), the second part covered media consumption, presenting 20 different series (15 with an autistic character or a character depicting autistic characteristics, five distraction items). For each series, participants had to indicate how much of the show they watched, how long ago they watched it and how much they liked the show. For all series they watched, participants had to rate how much they liked the selected character and give them a diagnosis or indicate whether they think they have no diagnosis. The third part investigated subjective opinion of the participant about their own health (depression, anxiety and autism) and participants were asked if they have an official diagnosis by a psychological professional. Lastly, an emotion recognition task (ERT) was done as an objective indicator for autism.

Participants were recruited online by distributing the questionnaire via the research group’s network and via a panel distribution of the Österreichische Gallup-Institut GmbH. This study was performed in line with the principles of the Declaration of Helsinki. Approval was granted by the Karl Landsteiner Commission for Scientific Integrity and Ethics (EK-Nr. 1004/2023). All participants gave informed consent at the beginning of the questionnaire by ticking a box indicating their consent.

### Materials

#### Series Depicting Autism

We examined media consumption of 15 mainstream series depicting characters with autism or autism-like symptoms (Table 1). We added five distraction items depicting a character with a different mental condition. Of the 15 included series, 10 characters were canonically autistic, meaning the diagnosis is explicitly mentioned in the series, whereas five characters only depicted characteristics of autism without an explicit diagnosis.

**Table 1:** Series Used in The Study

Series	Character
Atypical	Sam Gardner
Bones	Temperance Brennan
Stranger Things*	Will Byers
The Good Doctor	Shaun Murphy
BoJack Horseman*	BoJack
Elementary	Sherlock Holmes
The Big Bang Theory	Sheldon Cooper
Ella Schön	Ella Schön
13 Reasons Why*	Hannah Baker
The Bridge US	Sonya Cross
Community	Abed Nadir
Love On The Spectrum	Kaelynn
Mr. Robot*	Elliot Alderson
As We See It	Violet
The A-Word	Joe Hughes
Sherlock	Sherlock Holmes
Everything’s Gonna Be Okay	Matilda Moss
The Queen’s Gambit	Beth Harmon
Sex Education*	Aimee Gibbs
The Bridge SE	Saga Noren

**Note.** * Distraction items (series depicting a character with a mental illness that is not autism)

### Subjective and Objective Autism

For subjective autism, we used a single question “How strongly do you yourself feel affected by autism?” with a visual analogue scale ranging from 0 to 100. We used a short version of the ERT for an objective measure of autism (Kessels et al., 2014). We decided for this task rather than a self-administered questionnaire because difficulties in emotion recognition is a characteristic of autism that can be measured objectively. In the ERT, participants get presented with 48 different facial expressions. They need to decide if the face expresses surprise, happiness, sadness, fear, disgust or anger on four difficulty levels. We shortened the original 96 items measure by removing two faces (one male, one female) for all difficulty levels.

### Analysis

Our variable for media consumption was calculated by adding the values of the two questions for how much of the series the participants have watched and how long ago they have watched it (assuming the effect might not be stable over time). Valence was calculated for both how much participants liked the show and the character. To explore the association between self-diagnosed autism and media consumption, we calculated a linear regression analysis. A Pearson correlation coefficient was computed to assess the linear relationship between the subjective autism diagnosis and the ERT-results. Distraction items were excluded from the analysis.

## Results

### Participants

A total of 348 participants aged 18 to 30 participated in the study (mean age: 24.49 years, SD = 3.71). 117 participants identified as male, 213 as female and 17 as non-binary. Most of the sample was Austrian (n = 291). Specific data on socioeconomic status and educational attainment levels were not recorded. As we aimed for a general population, we neither specifically included nor excluded autistic people. Two participants indicated an official diagnosis for autism.

### Media and Self-Diagnosis

Regression analyses for all examined variables can be found in Table 2. Media consumption significantly predicted autism self-diagnosis, β = 0.122, *t*(335) = 2.292, *p* < .05. No other included variable predicted autism self-diagnosis. There was no significant correlation between autism self-diagnosis and ERT-scores as an indicator for an existing autism spectrum condition, r(346) = − 0.025, *p* = .648. There was no significant effect of gender-self-identification on autism self-diagnosis, *t*(328) = − 0.023, *p* = .818. The 17 people identifying as non-binary could not be analysed due to the small number.

**Table 2:** Regression Analysis

Variable	B	SE(B)	β	t	P
Intercept	9.954	9.021		1.103	0.271
Media consumption	0.301	0.137	0.122	2.192	0.029*
Positive valence character	-1.796	2.064	− 0.063	− 0.870	0.358
Positive valence series	0.983	2.167	0.034	0.454	0.650
ERT-Score	− 0.061	0.201	− 0.016	− 0.302	0.763

**Note**. *N* = 340. *p < .05. *R²* = 0.018

## Discussion

This study explored the link between series depicting autistic characters and self-diagnosis of autism. We found that the media consumption significantly predicted the self-diagnosis of autism. Self-diagnoses and ERT-scores, as an objective measure of autism-spectrum conditions, were not significantly correlated. Female and male participants rated themselves equally high on the self-diagnosis scale.

The effect we observed was small. This is to be expected as media consumption and portrayal of autism in series most likely only plays a small role in autism characteristics and diagnosis (Hansen et al., 2015; Huang et al., 2020; Zeidan et al., 2022). Yet, the results show that media consumption does relate to self-diagnosis. As such, it becomes even more important to accurately portray the diverse experiences of the autistic community in mainstream media. Interestingly, none of our control parameters was significant in our analysis, which means that self-diagnosis was only related to media consumption time, but not to objective diagnoses or participants’ subjective valence of the watched shows or portrayed characters. This means that the mere exposure to the media content might be sufficient to influence self-diagnosis unrelated to how much viewers liked the show or the autistic character.

### Implications for the Autistic Community

People who diagnosed themselves as autistic have not necessarily shown low scores on objective tests. In fact, some participants with the highest scores on the ERT (which means high ability in emotion recognition) also scored themselves very high on subjective measures of autism in our sample. This has implications for the autistic community because it suggests that some of the symptoms experienced by self-diagnosed individuals may rather be subjective than actually reflecting the symptoms of autism, which could result in a dilution of the challenges and specific experiences faced by people with an actual diagnosis of autism. Paradoxically, this could lead to less understanding of the autistic community, despite there being more awareness of the condition.

### Limitations and Future Directions

As this was an initial exploratory study around the phenomenon of media depicting autism and self-diagnosis in a general population with a rather simple research question, we did not include autistic people in the design of this study. As it is recommended to include the community members in the design of this kind of studies, this can be seen as a limitation. Furthermore, we tested subjective autism / self-diagnosing only with a single question.

Further research might examine the phenomenon on a more detailed level, for example in what ways people subjectively feel autistic. Considering that tic-like behaviours have a high possibility to elicit social contagion (Haltigan et al., 2023), repetitive behaviours might be self-diagnosed more likely after watching series than social difficulties. Lastly, future research could look into different types of media, such as social media, to gain further insight into media portrayal and self-diagnosis of autism.
